# Effectiveness of a Web-Based Intervention on Parental Psychological Flexibility and Emotion Regulation: A Pilot Open Trial

**DOI:** 10.3390/ijerph18062958

**Published:** 2021-03-13

**Authors:** Juan M. Flujas-Contreras, Azucena García-Palacios, Inmaculada Gómez

**Affiliations:** 1 Department of Psychology, University of Almeria, 04120 Almeria, Spain; igomez@ual.es; 2 Health Research Centre (CEINSA/UAL), University of Almeria, 04120 Almeria, Spain; 3 Department of Psychology, University Jaume I, 12071 Castellon, Spain; azucena@uji.es; 4 CIBER of Physiopathology of Obesity and Nutrition CIBERobn, CB06/03 Instituto de Salud Carlos III, 28029 Madrid, Spain

**Keywords:** contextual therapies, eHealth, emotional regulation, parenting, pilot trial, psychological flexibility, web-based intervention

## Abstract

“Parenting Forest” is an informed contextual therapy parenting program for improving parental emotion regulation strategies and psychological flexibility. The aim of this study was to evaluate the preliminary effectiveness of a self-guided web-based intervention of the Parenting Forest program. The intervention program consists of six self-applied sequential modules that use strategies from contextual therapies for providing a parenting style open to experience, mindful and committed to its actions. A pilot controlled open trial was conducted. Eligible parents (*n* = 12) enrolled in the web-based intervention completed baseline (T1) and post-intervention (T2) assessment instruments. Parental psychological flexibility, avoidance, emotional regulation, parental stress, satisfaction with life, children’s psychological adjustment and client satisfaction were measured to assess the effects of the intervention. Mood, coping, and value-related actions were assessed as measures of progress. The results showed positive effects on the parents’ psychological flexibility and emotion regulation. Parents’ mood and coping skills improved throughout the intervention program. These results provide preliminary evidence of the web-based Parenting Forest’s efficacy, although further research is needed to assess its effectiveness for prevention and in clinical populations.

## 1. Introduction

Parental practices have been shown to have a protective effect on family problems and their children’s psychological adjustment. Undemocratic parenting styles can lead to internalizing and externalizing problems and less prosocial behavior in childhood [1,2]. Authoritarian and controlling parental styles are risk factors for bullying [3], internalizing problems [4], anxiety, depression, and suicidal ideation [5]. Parental stress has also been shown to be a mediator of child behavioral problems [6] and leads to worse parenting practices related to psychological inflexibility [7].

Parental psychological flexibility is defined as the ability to be fully aware of the emotions and thoughts related to childcare, taking them in perspective, and redirecting behavior into parenting practices in line with values [8]. In contrast, experiential avoidance refers to attempts to reduce or eliminate events or stimuli that produce discomfort [9]. Experiential avoidance responses have been associated with problems of anxiety [9], stress [10], depression and family conflict [11] in both children and parents. Psychological inflexibility in parenting practices can lead to dysfunctional reactions or interactions as a result of cognitive fusion and avoidance of the emotional distress associated with parenting, i.e., avoiding children’s reactions, overprotective or impulsive parental reactions that occur with the function of trying to avoid confronting situations or private events (thoughts, emotions, feelings, sensations, etc.) that might cause discomfort [12,13,14,15]. Moreover, psychological flexibility has been related to emotion regulation [16] as an adaptive strategy, that is, strategies that are linked as a protective factor against psychopathology or are associated with a good health and well-being outcome for individuals [17]. Related to psychological flexibility in parenting, mindful parenting is defined as the use of mindfulness strategies, i.e., full attention in the present moment, to enable parents to redirect their attention to the needs and interactions with their children [18]. Promotion of psychological flexibility and mindful parenting are related to a better development of emotion expression [19], regulation skills [20], and mental health of children and adolescents [21]. These skills are mainly addressed by third-wave behavior therapies (or contextual therapies) that attempt to enhance skills as (a) acceptance and emotion regulation, (b) mindfulness or attention-focusing strategies, and (c) motivation to change towards values [22]. These are a heterogeneous group of therapies, that share common characteristics and psychological process, which emerged as an expansion of behavioral and cognitive–behavioral therapies [22].

The goal of parenting training programs is to improve skills that improve or facilitate a positive parenting style. Training in behavioral parenting has shown positive effects, especially for behavior problems, developmental disorders, and eating problems [23]. For example, behavioral parenting intervention has also been associated with improved family dynamics, problem solving and communication between family members [24]. Despite the strong evidence that it improves parenting and family relations, there are often problems in carrying out parenting intervention. Only about 35% to 50% of parents who enroll in interventions actually attend [25]. Lack of time, the burden of childcare, scheduling and transportation incompatibility [26], social stigma [27], or emotional barriers [13] are some of the factors that make it difficult to maintain treatment adherence. Adherence to technology-based parenting interventions increases by 83% [28].

In addition to these limitations in evidence-based intervention, the general population has little access to them [29]. Technology-based intervention is a promising method in clinical psychology, as a large number of people who seek help can attend without the limits of time or space [30,31], and with an accessible, brief and flexible format [32]. The potential of technology-delivered intervention for parents has been emphasized [33,34].

Technology-based parenting intervention has mainly been online [35], although computer programs, podcasts, and video series have also been used [36]. Web-based parenting intervention has shown parent–child outcomes with moderate effect sizes and a high level of satisfaction [35,37], and larger effect sizes for parents with children diagnosed with a medical illness [28]. As larger effect sizes have been found for treatment intervention than for prevention [28], different levels of support may be necessary to target family needs [38]. However, parents who have had access to universal prevention programs in parenting have indicated greater satisfaction and sense of competence than those who did not [39]. Online intervention for families has shown evidence of reducing children’s behavioral problems [40,41], preventing such affective problems as anxiety and depression [42], improving communication skills of children with autism spectrum disorder [43], and improving problem-solving by parents with children with cancer [44]. Web-based Parent–Child Interaction Therapy showed a larger effect size than online psychoeducation or cognitive–behavioral therapy-based parenting intervention [28].

Informed parenting intervention with third-wave therapies has mainly been implemented with Acceptance and Commitment Therapy (ACT) [45], Dialectical Behavior Therapy (DBT) [46], and Mindfulness [47]. A review of ACT for parents found that it has mainly been applied to parents of children with developmental disorders and ASD, chronic pain, physical, and mental health problems [48]. These interventions have shown positive effects on parental distress status, parental psychological flexibility, emotion regulation and improvement in children’s problems [48].

Mindful parenting intervention has shown positive effects in reducing stress, worry, improvement in parenting practices, and family functioning [49], emotional well-being and stress for parents of children with autism, for parents with children with disabilities [50], and on reactivity and stress in parents of children with ADHD [51]. The inclusion of mindfulness strategies in parenting training can be beneficial to family functioning, parent–child relationships, and parenting strategies [52].

DBT intervention for parenting is included in adaptation for children (DBT-C) and adolescents (DBT-A), with improvement in depressive symptomatology, behavioral problems and functioning [53]. Although these adaptations are child-oriented, they include a parenting module that aims to create a validating environment and influence behavior [54,55]. Other DBT-informed interventions, such as parental intervention with DBT strategies, have shown positive effects on emotion regulation, reduced affective symptoms, increased maternal acceptance, and parental practices [53].

Few studies have applied third-wave therapies to promoting parenting on the internet. A study by Sairanen et al. [56] of online ACT applied to parents of children with chronic medical conditions found large effect sizes in improving stress and mindfulness skills, as well as moderate effect size for improvement in depression and anxiety. A combination of ACT and ABA (applied behavior analysis) on the Internet for parents of children with ASD found improvement in parental knowledge and prosocial behavior and children’s hyperactivity [57]. Positive effects on mindfulness skills and depressive and anxiety symptoms have been found in pregnant mothers with online mindfulness intervention [58].

This pre-experimental study was a preliminary test of the effectiveness and satisfaction of a web-based parenting intervention protocol for promoting emotion regulation and parental psychological flexibility. It assessed the feasibility of an online clinical protocol based on an informed contextual therapy parenting program, using mainly ACT strategies, that has been tested in a face-to-face group format with positive results [59]. We hypothesized that a significant improvement would be found in the main variables, that is, parental psychological flexibility, emotion regulation, and parental stress. A resulting reduction in problems and difficulties of their children was also expected.

## 2. Materials and Methods

### 2.1. Design

This study employed a controlled, non-randomized, pre–post open trial. It received the ethical approval of the Andalusian Health Service’s Almeria Research Committee. The CONSORT 2010 Statement: extension to randomized pilot and feasibility trials [60] adapted to non-randomized studies was followed [61].

### 2.2. Participants

Parents were recruited by posting information on Facebook, at children’s mental health centers and parents associations in Almeria. The inclusion criteria were: (a) parent or legal guardian, (b) of a child from 3 to 16 years old (c) with difficulties in flexible parenting based on Parental Acceptance Questionnaire scores (6-PAQ) [62]; or (d) child with borderline or abnormal scores in emotional or behavioral difficulties, as measured by the Strengths and Difficulties Questionnaire (SDQ) [63]; (e) email and daily access by internet; (e) parent/guardian not diagnosed with any psychological or substance use disorder that would impede participation in the study; (f) no parent/guardian language or comprehension barriers.

The participants filled out an online form with their data. If they met the criteria for inclusion, they gave their informed consent before starting the study. Participants did not receive any financial incentive for study participation.

The pilot trial sample size was calculated in relation to the clinical trial [64]. Julious [65] recommends a group of 12 per experimental condition for pilot studies based on feasibility, precision of the mean and variance, and regulatory considerations. We would need a sample size of 12 to reliably (with probability greater than 0.7) detect an effect size of δ ≥ 0.8, assuming a two-sided criterion for detection that allows for a maximum Type I error rate of α = 0.05.

**Figure 1:**
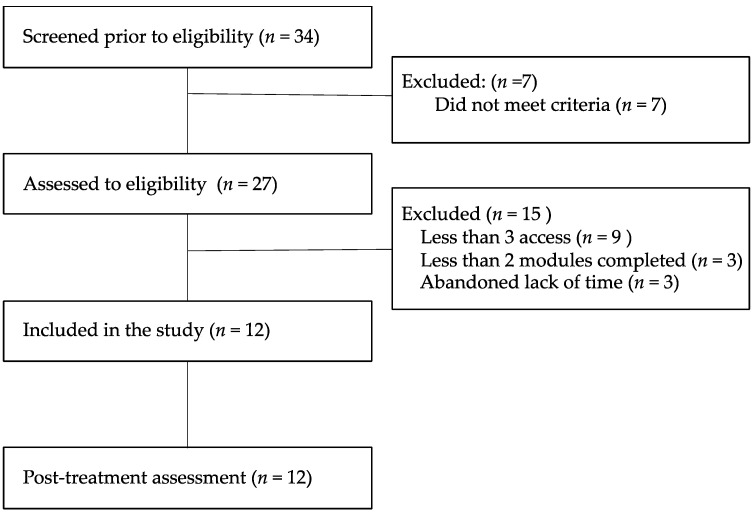
Flowchart of participants.

Figure 1 shows the participant flowchart. Of the 27 participants that met the study’s inclusion criteria, 15 were excluded for not accessing the platform, not completing the units, or withdrawing from treatment due to lack of time. Twelve participants who were included in this pilot study completed the intervention.

The incidental sample was comprised of 12 Spanish participants (fathers and mothers individually) aged 36 to 50 (*M* = 41.1; *SD* = 4.17), of whom 83.3% (*n* = 10) were women. A total of 66.7% (*n* = 8) of the parents were married, 1 widowed, 1 common-law couple and 2 divorced. Most parents had two children (66.7%; *n* = 8) aged six months to 15 years (*M* = 7.84; *SD* = 3.48). Table 1 presents the sociodemographic data and psychological characteristics of each participant.

### 2.3. Intervention

The online parenting intervention was carried out using the Psychology and Technology Platform [66]. This web platform has been successfully employed in previous clinical trials for emotional disorders [67]. When the participants had filled in their sociodemographic data and confirmed their informed consent, they were contacted by e-mail and given individual 24/7 access to the intervention on the Web platform with a username and password. Parents complete the program individually.

“The Parenting Forest” intervention protocol aims to promote emotion regulation skills and parental psychological flexibility. The components are based on third-wave therapies [13,15,68,69]. To achieve this goal, exercises and activities are proposed for: (a) fostering full awareness and attention to the present, both in parents’ private events and in their relationships with their children; (b) the use of verbal regulation strategies based on acceptance, as opposed to avoidance behavior and emotional suppression; (c) increasing perspective taking of self-thoughts, emotions or sensations (defusion); (d) providing an environment of emotional validation in relation to parenting and to oneself; (e) promoting behavioral activation in actions towards personal values, specifically related to their children. This protocol has previously been applied in a face-to-face group format with positive effects on parental psychological flexibility, parental stress and emotion regulation, and reduction in emotional and hyperactivity symptoms in their children [59].

The parenting intervention is organized in six sequential self-applied units. Participants are encouraged to take one unit a week and practice the homework the following week. The first four units focus on promoting personal and parenting skills. The last two units are focused on parent–child relationships. From the very beginning, the parents work on setting the clear individual values-related goals that will guide the treatment (Table 2). Unit contents are delivered as video instructions, metaphors and exercises, text and explanations about the content, and downloadable worksheets.

**Table 2:** Descriptions of the modules, components and contents included in the clinical protocol.

Module Title	Module Content
1. Welcome to the parenting forest	1. Introduction to clinical protocol: objectives and sequence. 2. The “Forest Metaphor”: introduction to work on actions towards values. 3. The mind is a lake metaphor: emphasizes that the thoughts and emotions form part of the mind, that they are there and are part of our nature. 4. Explanation of what is mindfulness and first practice of mindfulness to breathing. 5. The garden metaphor [70] values clarification and general goals. 6. Self-evaluation of contents and homework.
2. Emotion regulation with acceptance	1. Welcome and objectives of the module. 2. The “garden exercise”: Reminder of the contents of the last session. Establishment of specific goals and steps for actions in the direction of values. 3. The “shelter”: exercise of the wise mind [71]. Identify actions involving the emotional mind vs. the rational mind. Establish cohesion with the parenting and look for alternative reactions. 4. Self-evaluation of contents and homework.
3. Walking through the forest	1. Welcome and objectives of the module. 2. The “garden exercise”: evaluation of the achievement of actions. Identification of barriers and difficulties and alternative actions. 3. “Body scan” mindfulness exercise. Physical sensations related to emotions. 4. “The star observatory”: a defusion exercise to strengthen perspective-taking in private events. 5. Self-evaluation of contents and homework.
4. Allow your emotions to flow	1. Welcome and objectives of the module. 2. The “garden exercise”: evaluation of the achievement of actions. Identification or barriers. Generalization of parenting skills to goals. 3. “Full attention of sound” mindfulness exercise. 4. The “cascade of emotions”: deliteralization of private events. 5. Self-evaluation of contents and homework.
5. Analyzing your parenting	1. Welcome and objectives of the module. 2. The “garden exercise”: self-assessment of achievements of actions. Identification or barriers. 3. Functional analysis of parenting for of both parents’ and children’s behavior. Generalization of parenting skills applied to analyzed situation. 4. Thoughts are clouds exercise: deliteralization of private events. 5. The “Connect and Shape” parenting model [72]. 6. Self-evaluation of contents and homework.
6. The other side of the forest	1. Welcome and objectives of the module. 2. The “garden exercise”: evaluation of the achievement of actions. Empowerment for future goals. 3. Review of the “Connect and Shape” parenting model [72]. 4. Provide behavioral strategies for managing their children’s behavior and emotional problems: modeling, identification of reinforcers, reinforcement, control of antecedent stimuli, differential reinforcement, Premack principle, extinction, overcorrection and timeout. 5. Review skills that have been most useful and relapse prevention.

The participants received an automatic email after not accessing the platform for 15 days. Every 10 days, the researcher sent an e-mail to parents who had not accessed the platform for over a week to monitor the evolution of the intervention and to promote adherence to the treatment. Participants received an automatic email reinforcing actions toward parental values if they reported a score of seven or more on the consistency in actions toward values progress question. Participants were automatically emailed encouraging them to continue working on their goals if they reported a score of six or less.

After Unit 5, participants received a report on parental practices based on their results in the Parental Education Styles Questionnaire [73], which was completed at the end of the module. Participants could see their progress in a section called “How am I?”. In this section, they could see their pre- and post-flexible parenting scores, and their mood, coping and actions towards values progress scores. They could also access the modules again to review them after completion.

### 2.4. Measuring Instruments

#### 2.4.1. Instruments to Assess Primary Parental Outcomes

The Parental Acceptance Questionnaire (6-PAQ) [74] was applied to assess parental psychological flexibility and three related behavioral styles: An open response style to the experience with acceptance and a full and flexible willingness (Open). A style that is fully aware of the present moment and has a perspective on their private events (Aware). A style committed in its actions towards values that make sense to the person (Active). The Spanish version of the scale consists of 16 items on a four-point Likert scale. A higher score indicates greater parental psychological inflexibility. The Cronbach’s alpha is 0.81 [62]

The Acceptance and Action Questionnaire-II (AAQ-II) [75,76] measures experiential avoidance in 7 items on a 7-point Likert scale that reflects the tendency to avoid or escape from thoughts, sensations or emotions that produce discomfort. A higher score indicates greater experiential avoidance. The scale has a Cronbach’s alpha of 0.88.

The Difficulties in Emotion Regulation Scale (DERS) [77,78] was used to evaluate treatment effects on emotion regulation. This scale measures a total score and 5 emotion regulation processes in 28 items on a 5-point Likert scale. The instrument assesses emotional regulation difficulties related to lack of emotional awareness, poor emotional clarity, interference in goal-oriented behaviors, lack of acceptance strategies, and limited access to regulation strategies. A higher score indicates more difficulties in emotional regulation. The total score has a Cronbach’s alpha of 0.91.

The Parenting Stress Scale (PSS) [79,80] was used to asses stress related to parenting. This scale consisting of two dimensions in 12 items rated on a 5-point Likert-type scale: baby rewards, which refers to satisfaction with the parental role, and parent stressors, which refers to the level of stress related to parenting. A higher score indicates greater parental stress. The scale has a Cronbach’s alpha of 0.77.

The Satisfaction with Life Scale (SWLS) [81,82] was used to assess general satisfaction with life. It consists of 5 items on a 5-point Likert scale. The average score of the instrument is 24.16. A higher score indicates higher life satisfaction. The Cronbach’s alpha is 0.88.

#### 2.4.2. Measures of Parent’s Process Outcomes

At the beginning and at the end of each module mood, coping, importance of values (children as value), and consistency in valued actions was assessed. Mood was measured asking “How do you feel right now?” followed by a 5-face visual scale that has been used in previous studies [83]. Coping perception was assessed using the question “How able do you feel to address your concerns about your children right now?”. At pre-module, the importance and implication in valued direction actions towards positive parenting was assessed on a 10-point Likert scale (we have transformed this score to a 5-point scale to make it comparable to the other process outcome).

#### 2.4.3. Instrument to Assess Children Outcomes

The Strengths and Difficulties Questionnaire (SDQ) [63,84] was used to assess treatment effects in children. This is a screening scale used to detect psychological problems in childhood and adolescence (between 4 and 17 years old). It has scales for parents, teachers, and self-reporters from the age of 11. The scale consists of a total score and 5 factors: emotional symptoms, behavioral problems, hyperactivity, problems with peers and prosocial behavior. The cutoff points for borderline and clinical range are set at the 80th and 90th percentiles, respectively (except for the prosocial behavior scale which is reverse). The total scale has a Cronbach’s alpha of 0.77 and subscales vary between 0.64 and 0.85. The parent scale was used for this study and it was assessed at pre-test and post-test.

#### 2.4.4. Measure of Intervention Satisfaction

The Client Satisfaction Questionnaire (CSQ) [85,86] was used to assess general satisfaction with the treatment of parents. The instrument was designed to assess user satisfaction with the community and health services received. It consists of 8 items in a 4-point Likert scale. A higher score indicates greater satisfaction with the intervention. The scale has a Cronbach’s alpha of 0.90. This variable was measured in the post-test.

### 2.5. Statistical Analysis

The statistical R software Jamovi [87] was used for data analysis. First, a descriptive analysis of the variables evaluated pre–post intervention, and the process was carried out. Given the sample size, in order to explore the effects of the intervention, a nonparametric test was applied for two Wilcoxon T-related samples. The effect size was assessed with Cohen’s d test for nonparametric tests, considering a small effect size for scores of 0.1, medium effect for scores of 0.3, and large effect for scores of 0.5 or higher [88]. To estimate the clinical efficacy of the intervention, the reliable change index (RCI) was calculated through the Jacobson and Truax [89] method for the main variables, that is, parental flexibility, emotional regulation and stress. To calculate the cut-off points, the criterion of “c” proposed by Jacobson and Truax [89] was used. The cut-off point was determined from the post-treatment scores mean and standard deviation score of the participants (dysfunctional population) and scores of the normative population of the validation studies of the instruments (functional population). The score obtained represents the weighted midpoint between the mean of the functional and dysfunctional distributions. The participants were classified as “recovered” if their score in the post-test implies a change in the value of the RCI and if it is lower than the cut-off score. They were classified as “improved” if it represents a change in the RCI value but not in the cut-off score. It was classified as “no change” when it did not meet the criteria of the RCI value, even though there was an improvement in the mean scores. It was determined as “deteriorated” if the score represents an increase in the RCI value in a dysfunctional direction.

## 3. Results

### 3.1. Descriptive Analysis

In a descriptive analysis of participant scores, we found that the parents were above the cut-off point on all the general and parental psychological inflexibility variables. In emotion regulation skills, scores were high on emotional acceptance, interference in goals, and access to emotional regulation strategies. The DERS total score was above the cut-off point, as were the average stress scores (Table 3).

Child-related clinical scores were found for emotional symptoms, behavioral problems, problems with peers, and SDQ total difficulty score. The prosocial behavior score was borderline (Table 3).

In the post-test, all psychological flexibility scores, both general and parental, were down to below the cut-off point. The average post-test scores for emotional regulation and parenting stressors were also below the cut-off point, while the life satisfaction score increased two points (Table 3).

Child-related emotional symptoms and behavioral problem scores fell to borderline. The prosocial behavior score increased to above the cut-off point, although still borderline (Table 3).

### 3.2. Intervention Effects

Participants completed the intervention in a mean time of 64.2 days (SD = 20.5), with a duration of between 37 and 100 days between the start and end of the intervention. Table 3 shows the results of the outcomes for parents and children. A Wilcoxon signed rank test showed a significant decrease in parental psychological flexibility associated with a large effect size. There were also significant reductions with a large effect size associated with two of the 6-PAQ subscales—open and aware parenting response style. Due to the small sample size, we also considered marginally significant results. There were significant reductions in acceptance and rewards of parenting with moderate-to-large effect sizes. Child-related scores were significantly lower on behavioral problems and prosocial behavior, both with large effect sizes.

### 3.3. Significant of Clinical Change

**Figure 2:**
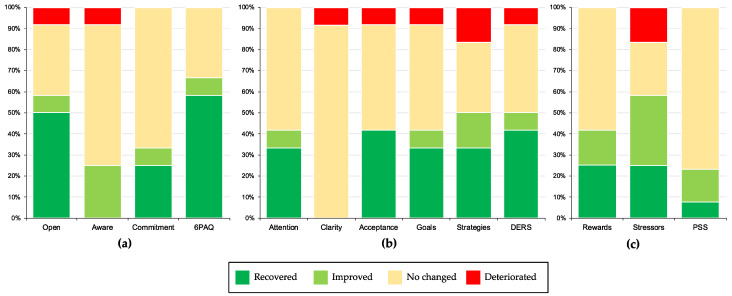
Percentages of participants recovered, improved, did not change, and deteriorated on (**a**) parental psychological flexibility; (**b**) difficulties to emotional regulation; and (**c**) parental stress.

The results for significant clinical improvements in main outcomes (a: parental psychological flexibility, b: emotion regulation, and c: parental stress) are summarized in Figure 2.

A functional change in parental psychological flexibility score was achieved by 67% of participants, of whom 58% recovered. Similar percentages were achieved in open response to experience (recovered: 50%; improved: 8%). Furthermore, 33% of the parents achieved a functional improvement in committed response style (recovered: 25%), and 25% of participants achieve a clinical improvement in centered-response style.

In emotion regulation, 50% of the participants achieved a functional improvement in emotional access to strategies (recovered: 34%; improved: 16%) and total emotion regulation scores (recovered: 41%; improved: 9%), 41% had a clinically significant recovery in acceptance skills, and 41% functional recovery in attention skills (recovered: 33%; improved: 8%), and goal-directed (recovered: 33%; improved: 8%) emotional regulation skills.

In parental stress, 58% of parents achieved a functional improvement in parenting stressors, of whom 25% recovered. Similarly, 41% of participants had a functional improvement in rewards of parenting (recovered: 25%; improved: 16%).

### 3.4. Process Outcomes

**Figure 3:**
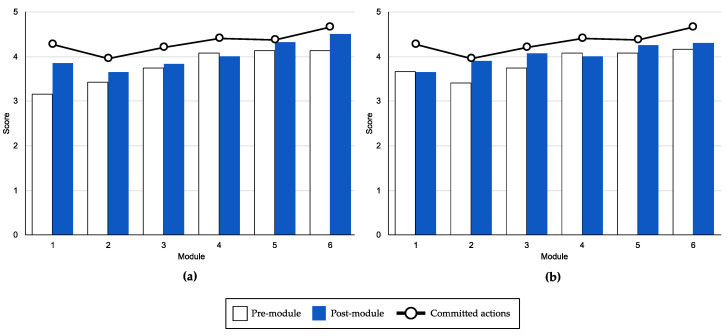
Process outcomes of pre-module and post-module scores of (**a**) mood and (**b**) coping.

Figure 3 shows the average scores on mood and coping before and after each unit. It shows that the scores at the end of the session were higher on both measures. There was an upward trend from 3.16 (*SD* = 0.9) to 4.5 (*SD* = 0.52) in mood and from 3.66 (*SD* = 0.9) to 4.66 (SD = 0.49) in coping. In Unit 1, the greatest difference between before (*M* = 3.16; *SD* = 0.9) and after (*M* = 3.83; *SD* = 0.93) scores was in mood. In coping, the largest difference between before (*M* = 3.41; *SD* = 0.9) and after (*M* = 3.9; *SD* = 0.6) scores was in Unit 2.

In actions committed to values (specifically to parenting), there was an upward trend from 4.26 (*SD* = 1.16) to 4.66 (*SD* = 0.65).

### 3.5. Measure of Intervention Satisfaction

The average score for satisfaction with the program was 28, which is considered average. Seventy-five percent of the participants believed that this program was very helpful in solving their problems and would strongly recommend it to someone they know. Similarly, in a descriptive analysis, 75% of the participants reported a high level of satisfaction, that is, scores between 3 and 4 for the items of the instrument. If it was necessary to seek help again, 75% of the participants said they would repeat it.

## 4. Discussion

The aim of this pilot study was to evaluate the preliminary effectiveness of and satisfaction with the “Parenting Forest” program, an online informed third-wave parenting training intervention to promote parental emotion regulation and psychological flexibility. This study examined the effects of the intervention on parental psychological flexibility, emotion regulation skills, parental stress, and satisfaction with life. Changes in the psychological adjustment of their children were also assessed.

The intervention achieved an improvement in parental psychological flexibility with a large effect size. Parental open to experience practices (Open) and awareness in the present moment (Aware) in particular increased with a large effect size. These changes are consistent with findings in the face-to-face group intervention in which large effect sizes were found for parental psychological flexibility, with open, aware and active response styles [59]. Web-based ACT intervention for parents of children with chronic conditions has shown improvement in mindfulness skills (related to Aware in this study) and cognitive defusion (related to Open) [56]. ACT online intervention has explored acceptance skills as a mediator of change in chronic diseases in adults [90,91]. Psychological acceptance has also been found to be a mechanism for change in the severity of mental health problems for parents of children with ASD [92].

In this pilot web-based intervention, no statistically significant changes in action and commitment to values (Commitment) were found. However, in the analysis of clinical changes, 33% of parents achieved a functional improvement. This small effect of the intervention can be explained by the lack of difficulties in this variable of parental psychological flexibility. Indeed, scores on this dimension were very close to the cut-off point at baseline. Furthermore, the self-reported progress scores on value-directed actions were also high from baseline.

No changes in experiential avoidance measures (i.e., psychological inflexibility as measured by the AAQ-II) were found in this study. These results are similar to those of previous web-based ACT parent studies [56], which suggests the importance of specific measures for more precise measurement of change in this population. Changes in parental psychological flexibility (measured with the 6-PAQ) in this study had a large effect size.

Acceptance-based emotion regulation skills and the rewards of parenting related to parental stress improved, but with a moderate effect size and lower statistical significance. In the analysis of clinical changes, the main effects were observed in access to adaptive strategies, acceptance skills, and the total score of emotion regulation skills. These changes are consistent with the face-to-face group parenting intervention, where large effect sizes were observed in acceptance skills at post-treatment, and in access to strategies and total DERS scores at follow-up [59]. Third-wave behavioral therapies for parenting have demonstrated positive effects on emotion regulation skills with DBT [93] and mindfulness strategies [94,95].

Parental stress has been related to parental psychological flexibility [7], so it is consistent to find improvements in these two variables in this intervention, although in this web-based intervention, it was found mainly in rewards of parenting. Previous studies of parenting interventions using mindfulness strategies have improved parental stress in parents of children with ADHD [96], intellectual disability [97], and ASD [98,99]. Moreover, mindfulness skills have a mediating effect on symptoms of depression, anxiety, burnout and stress in ACT online interventions for parents [100]. Improvement in parental stress was also found with intervention in mothers with emotion dysregulation using DBT strategies applied to parenting [93].

Child-related outcomes showed significant improvements in behavioral and prosocial behavior problems with a large effect size. Behavioral problems were found in a clinical range in most of the participating parents at the beginning of the intervention (eight out of the total), which could explain why large effect sizes were reported in this sample. A review of technology-based interventions for parents was more effective for problems than for their prevention [28]. Behavior problems have been improved in previous parenting programs such as the Online Triple P program [41] or parents with bipolar disorder [101]. Interventions for mindful parenting have been reported to reduce externalizing problems in children [102,103]. DBT-informed parenting interventions in multi-family settings have shown positive effects in reducing externalizing problems in adolescents [104].

Baseline prosocial behavior scores were clinical in four participants and borderline in seven. This was also one of the most common problems in the participating parents. However, the intervention was conducted in the context of the COVID-19 pandemic, when two of the main problems found in children were emotional symptoms and prosocial behavior [105]. Therefore, it could have be an incipient concern in parents, which was highlighted in this study. Mindfulness intervention in parents of teenagers with intellectual disabilities improved their children’s prosocial skills [106].

Progress showed an upward trend from the first module in mood and parental coping skills. After the modules, the post-module scores were higher than the pre-module scores. These data are consistent with those found in the face to face “Parenting Forest” group program [59]. The online intervention for positive parenting caused a progressive increase in mood from the first sessions [39]. Even though scores of actions were high from the beginning of the intervention, an ascending trend was still observed until the end, reaching a maximum of 4.66 points (on a 5-point scale). Previous studies using an ACT approach with parents of children with autism have shown progress in value-directed actions throughout the intervention [107]. From the ACT perspective, these values are differentiated from goals and increased task performance in students [108,109]. These effects might be transferable to other populations, such as parents.

Satisfaction with the program was high: 75% of the participants were very satisfied with the intervention. These data are consistent with previous studies that show a high level of satisfaction with online parenting programs [41,43,110].

One of the main problems identified in this intervention was the difficulty parents had in completing it. In this study, about 45% (12/27) completed the intervention. Only 12% explicitly left the study due to lack of time, while the rest continued with the intervention, but took longer than they should have. These attendance rates are similar to those found in face-to-face interventions [23,67], but lower than expected for online intervention [28]. We therefore want to explore usability and accessibility issues that would facilitate adherence to the treatment. Previous studies have shown that factors such as psychological distress, early help-seeking, or parental cognitions are important for adherence [111]. Future research would analyze the profiles of participants who did not complete it in comparison with those who did.

As a pilot study, it has a series of limitations that must be considered. First, the sample size is too small and located (Spanish) to allow generalization of the results or accurately detect the effects of treatment. However, this type of design provides a better ideographic analysis of the participants, which is useful for showing the preliminary results of the intervention. A second limitation is the lack of a control group, so the effects of the intervention cannot be isolated from other variables. To lessen this limitation, the effects of the intervention were compared with the preliminary group in the face-to-face intervention which used the same protocol. Thirdly, all the measures used are self-reported, which may have biased the data provided. The selection of participants has been made taking into account both parental and child factors, which may affect the outcome of the intervention, given that levels of impairment may be different at baseline. Finally, there is no medium or long-term follow-up of the effects of the intervention.

Although findings are promising and we are encouraged to continue in this line, this study is a preliminary trial. The limitations pointed out suggest future directions of research: (a) The need to conduct a larger study with a sample size large enough for a higher confidence interval in the effect sizes. Additionally, the wide age range of the children may affect the outcomes of the intervention, so future studies with larger sample sizes should consider differentiating the sample by age of the children (i.e., children vs. adolescents). (b) Assess usability and acceptability effects of the intervention to find out what variables can improve adherence to web-based intervention with this population. In addition, given that the intervention was carried out in a self-administered format, there is little control over intervention time. Therefore, in acceptability studies, it will be useful to explore how time may affect the outcomes of the intervention. (c) Comparative analyses with a control group for other intervention variables and follow-up of the results. (d) Given the differences found in the intervention effects between prevention and treatment in online interventions for parenting [59], it is important to consider cluster analyses of clinical versus community populations to check for differential effects.

## 5. Conclusions

To summarize, the results of the present pilot study suggest that the web-based parenting intervention is an alternative with positive effects on the main change variables. It shows the preliminary results of an intervention for parents based on third-wave therapies for improving parental psychological flexibility and emotion regulation. This protocol has already shown positive effects in face-to-face group intervention in families, so this study provides an alternative in an online format.

Parents involved in this pilot study significantly improved their parental psychological flexibility skills with a large effect size, specifically in openness to experience and greater awareness. Furthermore, improvements in emotion regulation skills with acceptance and greater perception of rewards of parenting were also achieved. This study also found reductions in problem behavior scores and increases in prosocial behavior of their children as a result of the intervention.

These promising results encourage us to further explore the effects of the intervention in a controlled clinical trial. In future research, the acceptability and usability of the online program will be explored. Although preliminary, the results of this pilot trial show high satisfaction of the participating parents.

## Figures and Tables

**Table 1:** Participants sociodemographic and psychological characteristics.

	Parent	Children	6-PAQ Parents Characteristics at Pre-Test	SDQ Children Characteristics at Pre-Test
1	42 (f)	10 (f) 7 (f)	↑ parental psychological inflexibility; ↓ acceptance; ↓ commitment	↑ SDQ score; ± emotional, ↑behavioral, ± hyperactivity, ↑peer, ↓ prosocial
2	37 (f)	8 (f)	↑ parental psychological inflexibility; ↓ acceptance; ↓commitment	↑ SDQ score; ↑ behavioral, ±hyperactivity, ↑ peer, ↓ prosocial
3	43 (f)	10 (m) 7 (f)	↑ parental psychological inflexibility; ↓ acceptance; ↓awareness	↑ SDQ score; ↑ emotional, ↑behavioral, ↑ hyperactivity, ↑peer, ↓ prosocial
4	37 (f)	3 (m)	± parental psychological inflexibility; ↓ acceptance; ↓awareness	↑ SDQ score; ± emotional, ↑behavioral, ± hyperactivity, ↑peer, ± prosocial
5	42 (f)	9 (m) 6 (m)	↑ parental psychological inflexibility; ↓ acceptance; ↓commitment	↑ SDQ score; ± emotional, ±peer./ADHD diagnosis < 6 months
6	36 (f)	9 (f) 13 (m)	↑ parental psychological inflexibility; ↓ acceptance; ↓awareness	↑ SDQ score; ↑ emotional, ↑behavioral, ↑ hyperactivity ADHD diagnosis > 1 year
7	40 (f)	7 (m) 3 (m)	-	↑ SDQ score; ± behavioral, ↑ peer
8	45 (m)	10 (m) 7 (m)	↑ parental psychological inflexibility	↑ SDQ score; ↑ emotional, ↑behavioral, ± prosocial
9	43 (m)	8 (m) 5 (m)/5 (f)	-	↑ SDQ score; ↑ emotional, ↑behavioral, ↑ peer
10	50 (f)	15 (m) 13 (m)	-	↑ SDQ score; ↑ emotional
11	42 (f)	10 (f)	-	↑ SDQ score; ↑ emotional, ±behavioral, ± peer, ± prosocial
12	36 (f)	8 (m) 4 (f)	↑ parental psychological inflexibility; ↓ awareness	↑ SDQ score; ↑ emotional, ↑behavioral, ± hyperactivity, ↓prosocial

Notes: (f): female; (m): male; ↑: high score; ↓: low score; ±: borderline score; <6 months: less than 6 months of diagnosis; >1 year: more than 1 year of diagnosis. The 6-PAQ range scores were calculated using the reliable change index criterion “c” described in Section 2.5. The SDQ range scores is obtained from the instrument’s validation study scales.

**Table 3:** Means (M), standard deviations (SD), Wilcoxon test and effect sizes (Cohen’s d) for outcomes of parents and children in the total score and factors of variables.

	Pre-Treatment (*n* = 12)		Post-Treatment (*n* = 12)		Wilcoxon Z	*p*	Cohen’s d
	M	SD	M	SD			
**Primary parental outcomes**							
6-PAQ	32.50	6.69	26.92	5.32	78.0	0.002 *	1.43
Open	11.17	3.13	8.67	1.92	50.5	0.021 *	0.91
Aware	12.42	3.00	10.00	2.73	45.0	0.009 **	1.39
Commitment	8.92	1.83	8.25	2.67	33.5	0.208	0.29
AAQ-II	23.17	7.87	19.33	8.76	49.5	0.154	0.49
DERS	66.00	24.73	57.33	18.33	53.5	0.272	0.38
Attention	8.58	3.92	8.58	2.50	24.0	0.905	0.00
Clarity	7.50	2.24	7.42	2.47	38.0	0.684	0.03
Acceptance	18.58	8.46	14.25	6.59	62.0	0.076 ^†^	0.57
Goals	11.17	4.32	9.92	4.68	45.0	0.304	0.29
Strategies	20.17	9.01	17.17	6.16	36.0	0.414	0.33
PSS	32.75	7.21	30.67	8.07	50.0	0.141	0.27
Rewards	8.92	3.40	7.83	2.82	44.5	0.089 ^†^	0.56
Stressors	23.83	5.57	22.83	5.77	34.0	0.540	0.15
SWL	23.25	7.94	25.08	5.58	27.0	0.365	0.31
**Children outcomes (reported by parents)**							
SDQ	23.75	6.97	23.25	6.82	23.5	0.478	0.16
Emotional sx.	4.00	2.41	3.58	2.27	28.0	0.545	0.24
Behavior prob.	4.83	2.72	3.75	2.80	28.0	0.018 *	0.71
Hyperactivity	4.75	3.14	5.25	3.49	15.5	0.433	0.28
Peers prob.	3.50	3.29	3.08	3.34	16.0	0.281	0.35
Prosocial	6.67	2.93	7.58	2.68	.003	0.034 *	0.84

Notes: ** *p* < 0.01; * *p* < 0.05; ^†^ *p* <0.10. 6-PAQ: Parental Acceptance Questionnaire; AAQ-II: Acceptance and Action Questionnaire; DERS: Difficulties for Emotion Regulation Scale; PSS: Parental Stress Scale; SWL: Satisfaction with Life Scale; SDQ: Strengths and Difficulties Scale; Sx.: Symptoms; Prob.: problems.

## Data Availability

The data presented in this study are available on request from the corresponding author. The data are not publicly available due to it is an open trial.
